# Clinical relevance of CompEx Asthma and impact on disease trajectory: benralizumab effect

**DOI:** 10.1183/23120541.00486-2025

**Published:** 2026-02-23

**Authors:** Clare Bolton, Praveen Akuthota, Njira Lugogo, Peter Barker, Thomas Bengtsson, Stefan Peterson, Salman Siddiqui, Carla A. Da Silva

**Affiliations:** 1 Early R&D, BioPharmaceuticals R&D, AstraZeneca, Cambridge, UK; 2 University of California San Diego, La Jolla, CA, USA; 3 Division of Pulmonary and Critical Care, Department of Medicine, University of Michigan, Ann Arbor, MI, USA; 4 Research and Development, AstraZeneca, Gaithersburg, MD, USA; 5 StatMind Statistical and Mathematical Modelling, Innovation and Design AB, Lund, Sweden; 6 National Heart and Lung Institute, Imperial College London, London, UK; 7 Early Respiratory and Immunology Clinical Development, BioPharmaceuticals R&D, AstraZeneca, Gothenburg, Sweden; 8 Digital Health Strategy and Implementation, Respiratory and Immunology, Evinova, Gothenburg, Sweden

## Abstract

**Background:**

Severe exacerbations (SevEx), the typical endpoint when evaluating asthma therapies, may provide incomplete assessment, as it relies on patient perception of disease and physician action. CompEx, a composite outcome that includes SevEx and acute worsening events (AWEs) (evaluated from e-diary entries using deterioration in peak expiratory flow (PEF), reliever medication use and worsening asthma symptoms), should provide more objective assessment. The correlation of CompEx event subtypes – SevEx only, AWE only or mixed SevEx/AWE – with disease trajectory and effect of benralizumab in the SIROCCO and CALIMA trials were evaluated.

**Methods:**

This was a *post hoc* analysis of patients (aged ≥12 years) with severe, uncontrolled asthma treated with benralizumab 30 mg or placebo every 8 weeks. PEF, symptoms and reliever medication use around CompEx event subtype occurrence, forced expiratory volume in 1 s (FEV_1_) trajectories and patient-reported outcomes were evaluated.

**Results:**

953 patients were included (benralizumab, n=465; placebo, n=488). Greater increases in asthma symptoms and reliever medication use, declines in PEF and slower return to baseline were seen around AWE and mixed SevEx/AWE than SevEx, according to treatment utilisation. Overall, patients without a CompEx event had the best FEV_1_ trajectory and patient-reported outcomes, compared with those with any CompEx event. Benralizumab reduced SevEx risk in patients experiencing SevEx only or mixed SevEx/AWEs; no effect was seen in patients with AWE only.

**Conclusions:**

CompEx includes SevEx and AWEs, both of which are clinically relevant events, providing a more comprehensive assessment of asthma worsening than SevEx alone. AWEs are particularly important contributors to poor asthma outcomes and should not be ignored when evaluating treatments.

## Shareable abstract

**The use of CompEx as an endpoint has shown clinical relevance by including not only severe exacerbations but also acute worsening events, both of which impact asthma outcomes and should be considered when evaluating treatments** https://bit.ly/4g5JYjJ

## Introduction

All patients with asthma are at risk of severe exacerbations (SevEx), including those with well-controlled disease [1]. This risk persists because episodic exposure to triggers can lead to rising inflammation and increasing symptoms, which may not always be fully addressed by maintenance therapy [2–4]. According to the American Thoracic Society and the European Respiratory Society (ERS) guidelines, SevEx are events that require immediate action, which may include the administration of systemic corticosteroids (SCS), an increase from a stable maintenance dose of SCS for a minimum of 3 days or a hospitalisation or emergency department visit due to asthma necessitating SCS treatment [5]. SevEx are typically used as the primary endpoint for clinical trials evaluating asthma treatments. However, this definition does not include critical loss of control events that are not treated, and it also relies on patient perception of symptoms and patient and physician assessment of the need for SCS.

Owing to the potential variability in SevEx as a result of under-reporting for some patients with asthma and over-reporting in those with a high symptom prevalence and better access to healthcare professionals, a number of composite endpoints have been proposed to better reflect patient experience, but these have not all been fully characterised or validated [6–11]. CompEx is one of these composite endpoints and was developed for the assessment of asthma treatments in clinical trials [12]. CompEx evaluates both SevEx and acute worsening events (AWEs), which are evaluated from diary entries recording deteriorations in peak expiratory flow (PEF), increased reliever medication use and/or worsening of asthma symptoms, including shortness of breath, cough, wheezing and chest tightness [12]. AWEs are recorded when either 1) at least two diary variables (one of which must be PEF) reach a prespecified threshold change from baseline over at least 2 consecutive days or 2) worsening greater than a certain magnitude (slope; daily rate of change) in all diary card variables occurs over ≥5 days, combined with a threshold change of at least one variable [12]. AWEs capture clinically meaningful worsening of asthma, but as they do not always result in SCS treatment they are not typically recorded in clinical trials unless they are associated with a SevEx. However, as AWEs are often self-managed, they have an impact on patients, contributing to poor health outcomes and reduced health-related quality of life.

CompEx has been retrospectively evaluated in comparison with SevEx using data from a number of clinical trials in asthma [13, 14]. A previous *post hoc* analysis of pooled 12-month data for benralizumab from the SIROCCO (n=768) [15] and CALIMA (n=844) [16] trials in patients with severe, uncontrolled asthma found that benralizumab reduced the risk of CompEx events, compared with placebo, to a similar degree as SevEx and AWE evaluated separately (risk ratio of 0.61, 0.60 and 0.59, respectively) [14]. In this analysis, the event rate ratios displayed similar patterns regardless of the baseline eosinophil count. However, it is worth noting that greater treatment effects were observed among patients with higher blood eosinophil counts [14]. The event rates were higher with CompEx than with SevEx due to the inclusion of AWE, which increased the power of the endpoint to detect differences between study arms.

In this *post hoc* analysis of data from the SIROCCO and CALIMA trials, we aimed to gain a greater understanding of CompEx as an endpoint by evaluating the different subtypes of CompEx event (*i.e.* SevEx, AWE or a mixture of SevEx and AWE) and their relationship to or influence on the trajectories of lung function, symptoms and quality of life over time. The impact of treatment with benralizumab on CompEx subtypes and disease trajectories (evaluated using forced expiratory volume in 1 s (FEV_1_), the 6-item Asthma Control Questionnaire (ACQ-6) and the Asthma Quality of Life Questionnaire (AQLQ)) was also evaluated.

## Methods

### Study design and data collection

This *post hoc* analysis evaluated pooled data from the 48-week SIROCCO [15] and 56-week CALIMA [16] phase III clinical trials, both assessing the efficacy of benralizumab *versus* placebo. Patients aged ≥12 years with a baseline eosinophil count ≥300 cells·µL^−1^ and severe, uncontrolled asthma were randomised 1:1:1 to benralizumab 30 mg once every 4 weeks, 30 mg once every 8 weeks (Q8W) or matching placebo. Both studies have been described previously [15, 16], and were conducted in compliance with the Declaration of Helsinki, the International Conference on Harmonisation of Technical Requirements for Registration of Pharmaceuticals for Human Use, the Good Clinical Practice guidelines and the ethics committee at each participating site. All participants or their legal representatives provided written informed consent.

We included data for patients from the benralizumab 30 mg Q8W (the currently approved dose [17]) and placebo arms of the SIROCCO [15] and CALIMA [16] trials to assess and compare the magnitude of response between the different subtypes of CompEx events and the effect of benralizumab on disease trajectory.

### Definitions of SevEx, AWE and CompEx events

In accordance with SIROCCO and CALIMA, SevEx events were defined as necessitating ≥3 days of SCS treatment, hospitalisation or an emergency department visit due to asthma necessitating SCS treatment [15, 16]. The algorithm to calculate an AWE has been described previously [12] and encompasses deterioration in PEF, reliever medication use and/or worsening asthma symptoms, with values collected from morning and evening patient e-diary entries (see supplementary material). Patients were considered to have had an AWE if at least two diary variables (with one being PEF) reached a predefined threshold change from baseline over a minimum of 2 consecutive days or if worsening exceeded a specified magnitude (slope; daily rate of change) across all diary card variables for a duration of ≥5  days, in conjunction with a threshold change in at least one variable.

**FIGURE 1:**
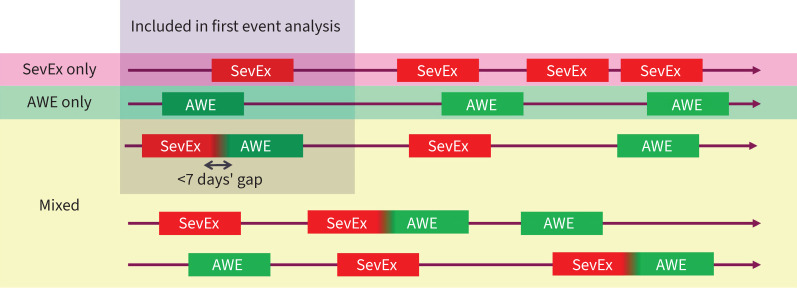
CompEx event types. SevEx: severe exacerbation; AWE: acute worsening event.

Patients were categorised into one of the following subgroups based on the occurrence of CompEx event subtypes throughout the study: 1) patients who experienced only SevEx during the trials, referred to as SevEx only; 2) patients who experienced only AWEs during the trials, referred to as AWE only; and 3) patients who experienced both SevEx and AWEs during the trials, referred to as mixed (figure 1). Within the mixed group, for the analysis according to first event type, including analysis of recovery after an event, patients who had a concomitant SevEx/AWE as the first event type were evaluated, but all other patients in the mixed group were excluded. Events starting or ending within 7 days of each other were considered a single event, with SevEx events accompanied by an AWE (or *vice versa*) within 7 days considered concomitant. For the analysis of the impact of event types on asthma variables, the groups analysed were SevEx only; AWE only; mixed events; any CompEx, which included all patients in the SevEx only, AWE only and mixed groups; and no CompEx, which included all patients who did not have a SevEx, an AWE or a concomitant SevEx/AWE event during the trials.

### Characterisation of the relationship between CompEx event types and disease trajectory and outcomes

In this study, we aimed to describe PEF, symptoms and reliever medication use in patients experiencing different CompEx event subtypes (SevEx only, AWE only or mixed). We also assessed recovery of PEF, symptoms and reliever medication use to baseline values 14 days after an event in patients experiencing different CompEx event subtypes. The relationships between CompEx event subtypes and FEV_1_ and between CompEx event subtypes and patient-reported outcomes were also evaluated. Patient-reported outcomes were assessed using ACQ-6 and AQLQ independent of treatment and following treatment with benralizumab 30 mg Q8W or placebo.

### Statistical analysis

The frequencies of CompEx subtypes (SevEx only, AWE only or mixed) were summarised using descriptive statistics and illustrated using Kaplan–Meier curves showing the number of patients at risk. CompEx subtypes were compared between treatments for time to first event using a Cox proportional hazards model adjusting for treatment, study, region, previous exacerbations and concomitant SCS use. Trajectories of FEV_1_, ACQ-6 and AQLQ were plotted according to whether or not a patient experienced a CompEx event, and according to CompEx subtype experienced (SevEx only, AWE only or mixed); data were imputed using last observation carried forward prior to calculating means. Changes from baseline in FEV_1_, ACQ-6 and AQLQ at weeks 24 and 48 in patients with different CompEx event types *versus* those with no CompEx events were evaluated using separate ANCOVA models with event type as a factor and baseline as a covariate. Profiles of diary card variables surrounding first CompEx event (SevEx only, AWE only or concomitant SevEx/AWE in the mixed group) were described using raw means without imputation. Sustained recovery was defined as having 2 consecutive days with values reaching at least the mean value calculated in the second week before the event (days −13– −7) in the 14 days after an event. If sustained recovery was not achieved within this time but the last available value was greater than or equal to the mean value calculated for the second week before the event, then the patient was defined as having achieved sustained recovery. Otherwise, patients not achieving sustained recovery were censored at day 14 or at the last day with data, whichever came first. Time to first day of sustained recovery was described using Kaplan–Meier curves showing the number of patients at risk. Homogeneity between patient groups of different CompEx event types in sustained recovery up to day 14 based on PEF, asthma symptoms and reliever medication use was evaluated by a Cox proportional hazards model and presented using pairwise hazard ratios with 95% confidence intervals. Further details of the analysis are provided in the supplementary material.

## Results

### Patients

A total of 953 patients aged ≥12 years with a baseline eosinophil count ≥300 cells·µL^−1^, receiving either benralizumab 30 mg Q8W (n=465) or placebo (n=488), were included. Baseline demographics for the pooled analysis population are shown in table 1. The mean (sd) ages were 47.9 (14.1) and 47.7 (14.7) years for patients receiving benralizumab and patients receiving placebo, respectively. The proportion of females was similar between patients receiving benralizumab (61%) and those receiving placebo (62%). Across the two studies, 214 (40%) of patients who experienced a CompEx event had SevEx only; 111 (21%) had AWE only and 204 (39%) had mixed events. Of the 204 patients who had mixed events, 65 (32%) had a concomitant SevEx/AWE event as their first event, and 139 (68%) had either a SevEx or an AWE as their first event. Baseline demographics for patients according to the type of CompEx event experienced are shown in table 2. E-diary compliance rates in CALIMA and SIROCCO were 94.2% and 91.3%, respectively.

**TABLE 1:** Baseline characteristics in the combined CALIMA and SIROCCO trials

Baseline characteristics	Benralizumab (n=465)	Placebo (n=488)
**Age, years**	47.9 (14.1)	47.7 (14.7)
**Female sex, n (%)**	283 (61.0)	305 (62.5)
**Age at diagnosis, years**	30.0 (18.2)	28.8 (18.8)
**Number of previous exacerbations per year, n (%)**		
** 2**	278 (60.0)	286 (59.0)
** 3**	107 (23.0)	110 (22.5)
** 4**	39 (8.0)	43 (9.0)
** ≥5**	41 (9.0)	49 (10.0)
**Use of maintenance OCS, n (%)**	68 (15.0)	67 (14.0)
**FEV_1_, % pred**	57.59 (15.30)	56.59 (15.00)
**FVC, % pred**	77.91 (15.70)	76.53 (15.30)
**FEV_1_/FVC, %**	59.94 (12.80)	59.98 (12.60)
**PEF, L·min^−1^**	281.1 (102.40)	268.5 (105.60)
**ACQ-6 score**	2.789 (0.90)	2.841 (0.96)
**AQLQ score**	3.940 (1.01)	3.939 (1.05)
**Blood eosinophil count, cells·µL^−1^, median (range)**	655.8 (300–2870)	668.4 (300–4150)

Data are presented as mean (sd) unless otherwise stated. OCS: oral corticosteroid; FEV_1_: forced expiratory volume in 1 s; FVC: forced vital capacity; PEF: peak expiratory flow; ACQ-6: 6-item Asthma Control Questionnaire; AQLQ: Asthma Quality of Life Questionnaire.

**TABLE 2:** Baseline characteristics for patients with composite endpoint (CompEx) events according to the type of event experienced

Baseline characteristics	SevEx only (n=214)		AWE only (n=111)		Mixed events (n=204)	
	Benralizumab (n=97)	Placebo (n=117)	Benralizumab (n=56)	Placebo (n=55)	Benralizumab (n=61)	Placebo (n=143)
**Age, years**	45.4 (15.3)	49.8 (14.6)	50.4 (12.2)	47.4 (13.7)	48.2 (14.6)	50.2 (12.0)
**Female sex, n (%)**	46 (47.4)	70 (59.8)	33 (58.9)	34 (61.8)	46 (75.4)	104 (72.7)
**Age at diagnosis, years**	29 (19.3)	31.2 (18.6)	32.1 (16.5)	28.7 (19.2)	25.7 (19.0)	30.3 (18.1)
**Number of previous exacerbations per year, n (%)**						
2	57 (58.8)	61 (52.1)	35 (62.5)	37 (67.2)	31 (50.8)	63 (44.1)
3	24 (24.7)	33 (28.2)	13 (23.2)	12 (21.8)	13 (21.3)	34 (23.8)
4	5 (5.1)	9 (7.7)	–	–	10 (16.4)	21 (14.7)
≥5	6 (6.2)	6 (5.1)	–	–	7 (11.5)	22 (15.4)
**Use of maintenance OCS, n (%)**	13 (13.4)	15 (12.8)	8 (14.3)	6 (10.9)	8 (13.1)	29 (20.3)
**FEV_1_, L**	1.813 (0.68)	1.634 (0.58)	1.704 (0.63)	1.716 (0.56)	1.592 (0.63)	1.587 (0.55)
**FVC, L**	3.106 (1.02)	2.790 (0.84)	2.876 (0.84)	2.884 (0.81)	2.733 (0.87)	2.690 (0.83)
**FEV_1_/FVC, %**	58.62 (12.4)	58.86 (12.5)	59.19 (13.9)	59.63 (10.7)	58.39 (14.3)	59.05 (13.1)
**PEF, L·min^−1^**	276.7 (103.9)	260.5 (86.1)	271.7 (111.7)	262.8 (109.0)	254.7 (85.9)	261.1 (97.4)
**ACQ-6 score**	2.856 (0.87)	2.910 (0.97)	2.753 (0.93)	2.873 (0.95)	2.954 (0.98)	2.838 (0.95)
**AQLQ score**	3.919 (0.91)	3.785 (1.04)	4.093 (1.09)	3.815 (0.95)	3.782 (1.05)	3.973 (1.03)
**Blood eosinophil count, cells·µL^−1^, median (range)**	510.0 (300–2700)	580.0 (300–2220)	525.0 (300–2370)	450.0 (300–3460)	500.0 (300–2110)	560.0 (300–3580)

Data are presented as mean (sd) unless otherwise stated. Patients in the SevEx-only group experienced only SevEx throughout the trial. Patients in the AWE-only group experienced only AWEs throughout the trial. Patients in the mixed events group experienced both AWEs and SevEx during the trial, including concomitant SevEx/AWE. SevEx: severe exacerbation(s); AWE: acute worsening event; OCS: oral corticosteroid; FEV_1_: forced expiratory volume in 1 s; FVC: forced vital capacity; PEF: peak expiratory flow; ACQ-6: 6-item Asthma Control Questionnaire; AQLQ: Asthma Quality of Life Questionnaire.

### PEF, symptoms and reliever medication use around the occurrence of different CompEx subtypes

**FIGURE 2:**
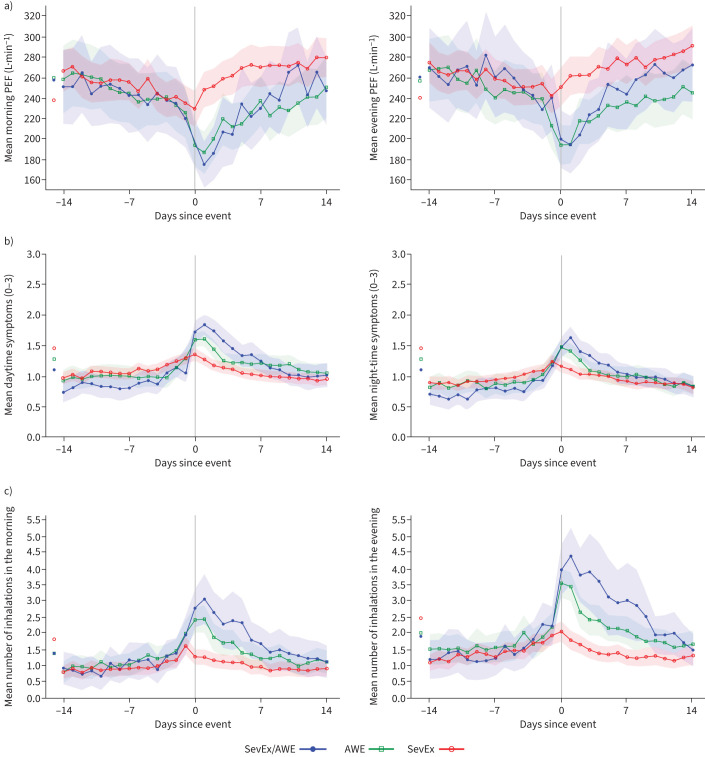
Morning/daytime (left) and evening/night-time (right) changes in a) peak expiratory flow (PEF), b) asthma symptoms (measured by the 6-item Asthma Control Questionnaire (ACQ-6)) and c) reliever medication use around the occurrence of a CompEx event in the combined CALIMA and SIROCCO trials. The three groups analysed were first severe exacerbation (SevEx) in the group that experienced SevEx only, first acute worsening event (AWE) in the group that experienced AWEs only and concomitant SevEx/AWE when it was the first event experienced in the group reporting mixed events. The shaded region shows the 95% confidence intervals.

Greater declines in morning and evening PEF were seen around the occurrence of a first event for patients with AWE only, and those with concomitant SevEx/AWE in the mixed group, than for those with SevEx only, as shown by the non-overlapping confidence intervals (figure 2a). Return to baseline PEF post-event was slower for patients experiencing AWE only and SevEx/AWE than for those experiencing SevEx only. Increases in night-time and daytime asthma symptoms (figure 2b) and reliever medication use (figure 2c) were greater for patients with AWE only and those with SevEx/AWE than for those with SevEx only. When analysed according to treatment received, although the sample sizes were smaller, there was no apparent difference between treatments and the pooled data in the magnitude of the deteriorations observed (see supplementary figure 1).

### Recovery of PEF, symptoms and reliever medication use after different CompEx subtypes

**FIGURE 3:**
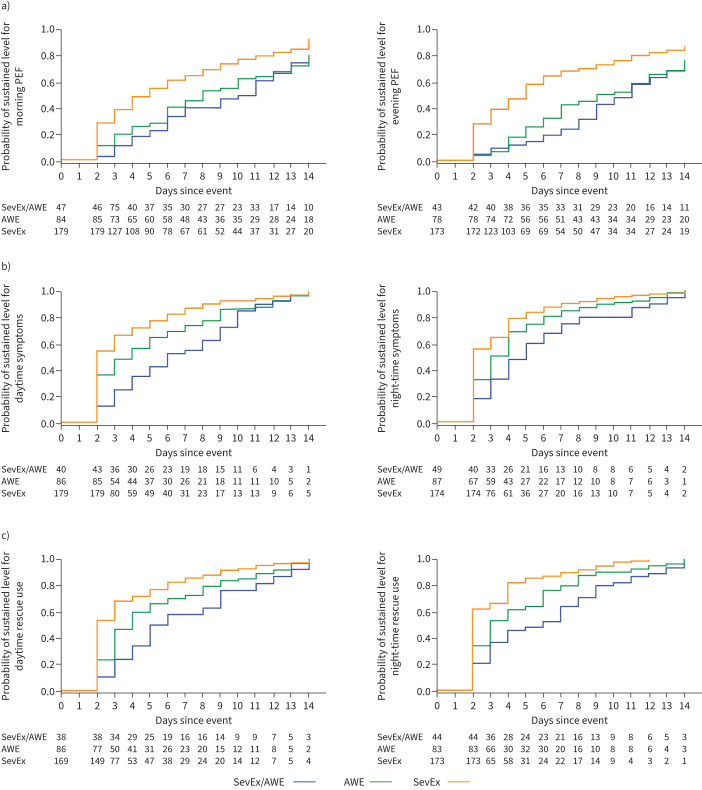
Recovery following a CompEx event to mean baseline values of a) morning (left) and evening (right) peak expiratory flow (PEF), b) daytime (left) and night-time (right) asthma symptoms and c) daytime (left) and night-time (right) reliever medication use in the combined CALIMA and SIROCCO trials. The three groups analysed were first severe exacerbation (SevEx) in the group that experienced SevEx only, first acute worsening event (AWE) in the group that experienced AWEs only and concomitant SevEx/AWE when it was the first event experienced in the group reporting mixed events. Day 0 was the first day of the analysed event. The numbers at risk for each analysis group at each time point are shown under each graph.

After 14 days post-event, in the analysis of sustained events according to first event type, the proportion of patients recovering to mean baseline morning and evening PEF was greater following SevEx (in the SevEx-only group: 89% and 89% for morning and evening PEF, respectively) than following AWE (in the AWE-only group: 79% (p<0.001) and 74% (p=0.001), respectively) or concomitant SevEx/AWE (in the mixed group: 79% (p<0.001) and 74% (p=0.003), respectively) (figure 3a; supplementary table 1). On average, recovery was slowest following a concomitant SevEx/AWE*.* Night-time and daytime asthma symptoms (figure 3b; supplementary table 1) and reliever medication use (figure 3c; supplementary table 1) returned to baseline levels for nearly all patients who experienced any event*.* As with PEF, recovery was slowest for patients who experienced a concomitant SevEx/AWE. When analysed according to treatment received, although the sample sizes were smaller, there was no apparent differences in recovery of PEF, symptoms or reliever medication use after different CompEx subtypes between treatments and the pooled data (supplementary figure 2).

### Efficacy of benralizumab on the CompEx subtypes

Compared with placebo, benralizumab showed efficacy in reducing events among patients experiencing SevEx only (hazard ratio (HR) 0.70, 95% CI 0.53–0.91; p=0.0084) or mixed events (HR 0.64, 95% CI 0.47–0.87; p=0.0041) throughout the study. Benralizumab did not reduce the risk of an event in patients who experienced AWE only (HR 0.92, 95% CI 0.64–1.34; p=0.6741; supplementary table 2).

### Relationship of CompEx with FEV_1_

**FIGURE 4:**
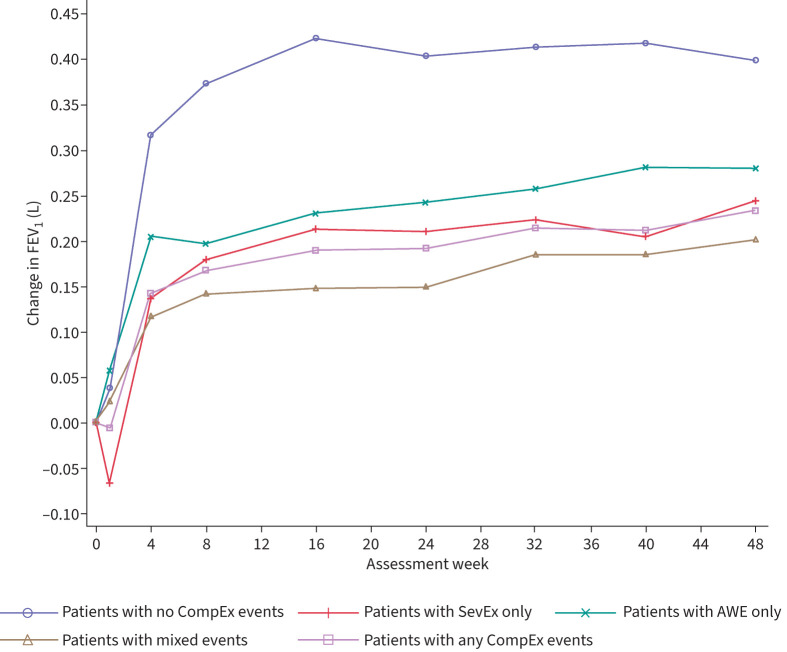
Forced expiratory volume in 1 s (FEV_1_) trajectory over time in the overall population of the CALIMA and SIROCCO trials. Data were analysed according to the type of event recorded: severe exacerbation (SevEx) only throughout the trial; acute worsening event (AWE) only throughout the trial; mixed events, including concomitant SevEx/AWE, throughout the trial; no CompEx events (*i.e.* no SevEx or AWE or concomitant SevEx/AWE) throughout the trial; and any CompEx event throughout the trial (this included all patients in the SevEx only, AWE only and mixed groups).

**FIGURE 5:**
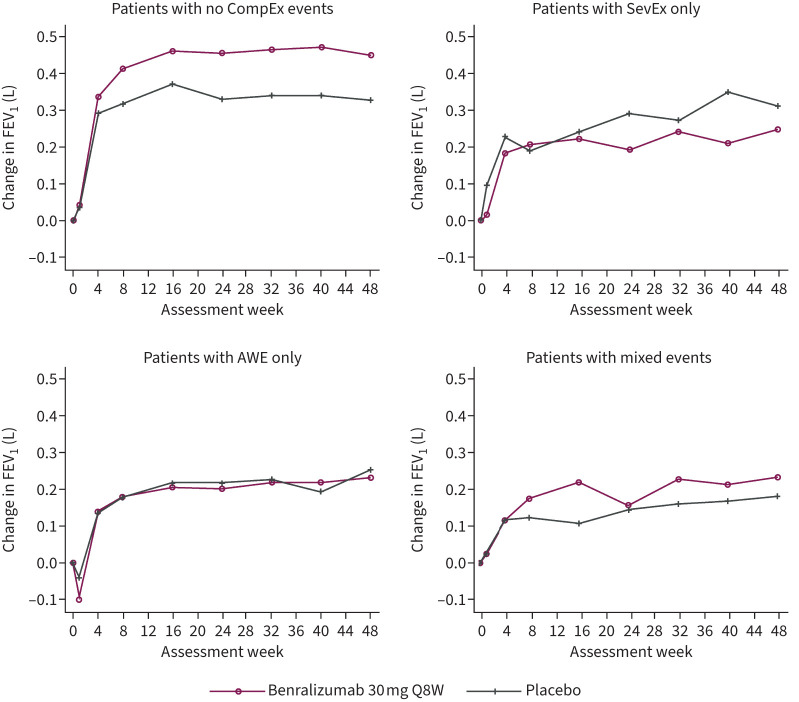
Forced expiratory volume in 1 s (FEV_1_) trajectory over time in patients treated with benralizumab or placebo in the combined CALIMA and SIROCCO trials. Data were analysed according to the type of event recorded: severe exacerbation (SevEx) only throughout the trial; acute worsening event (AWE) only throughout the trial; mixed events, including concomitant SevEx/AWE, throughout the trial; and no CompEx events (*i.e.* no SevEx or AWE or concomitant SevEx/AWE) throughout the trial. Q8W: once every 8 weeks.

Overall, patients who did not experience a CompEx event had the best FEV_1_ trajectory outcome over time, compared with patients who experienced any CompEx event (figure 4). Compared with patients who did not experience a CompEx event, the changes in FEV_1_ from baseline to week 48 were −0.154 L (p=0.0005), −0.119 L (p=0.0472) and −0.198 L (p<0.0001) for patients who experienced SevEx only, AWE only and mixed events, respectively (supplementary table 3). When considered according to treatment group, patients receiving benralizumab with no CompEx events (n=251) received the greatest benefit on FEV_1_ trajectory compared with those receiving placebo (n=173) (figure 5); an effect was also seen in patients experiencing mixed events, but no effect was observed in those experiencing AWE only or SevEx only.

### Relationship of CompEx with patient-reported outcomes

**FIGURE 6:**
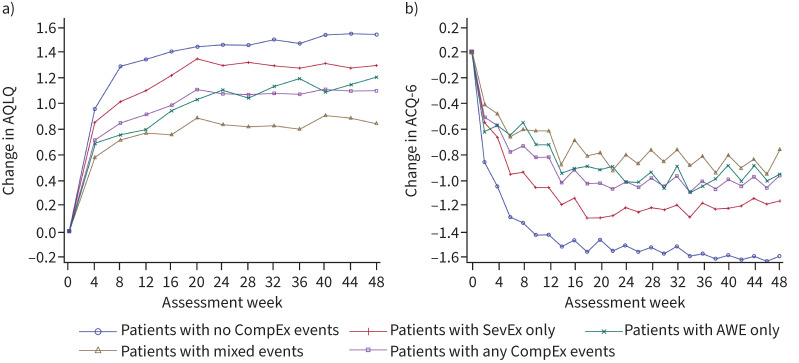
a) Asthma Quality of Life Questionnaire (AQLQ) and b) 6-item Asthma Control Questionnaire (ACQ-6) trajectories over time in the overall population in the combined CALIMA and SIROCCO trials. Data were analysed according to the type of event recorded: severe exacerbations (SevEx) only throughout the trial; acute worsening events (AWE) only throughout the trial; mixed events, including concomitant SevEx/AWE, throughout the trial; no CompEx events (*i.e.* no SevEx or AWE or concomitant SevEx/AWE) throughout the trial; and any CompEx event throughout the trial (this included all patients in the SevEx only, AWE only and mixed groups).

**FIGURE 7:**
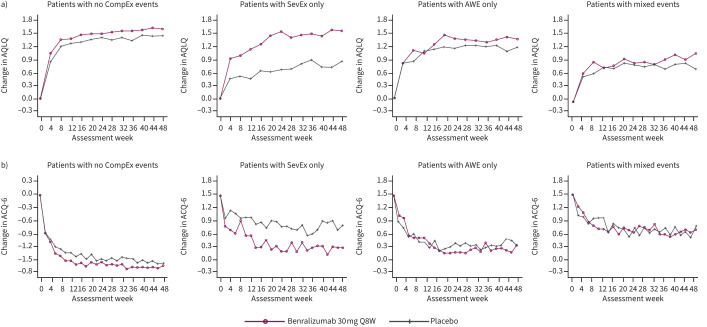
a) Asthma Quality of Life Questionnaire (AQLQ) and b) 6-item Asthma Control Questionnaire (ACQ-6) trajectories over time among patients treated with benralizumab or placebo in the combined CALIMA and SIROCCO trials. Data were analysed according to the type of event recorded: severe exacerbation (SevEx) only throughout the trial; acute worsening event (AWE) only throughout the trial; mixed events, including concomitant SevEx/AWE, throughout the trial; and no CompEx events (*i.e.* no SevEx or AWE or concomitant SevEx/AWE) throughout the trial. Q8W: once every 8 weeks.

Overall, AQLQ and ACQ-6 trajectories were greatest for patients who did not experience a CompEx event (figure 6). Patients who experienced mixed events had the poorest trajectories out of all groups evaluated. Compared with patients who did not experience a CompEx event, the changes in ACQ-6 from baseline to week 48 for patients who experienced SevEx only, AWE only and mixed events were 0.300 (p=0.001), 0.616 (p<0.0001) and 0.827 (p<0.0001), respectively (supplementary table 4). Compared with patients who did not experience a CompEx event, the changes in AQLQ from baseline to week 48 for patients who experienced SevEx only, AWE only and mixed events were −0.159 (p=0.1176), −0.419 (p=0.002) and −0.675 (p<0.0001), respectively (supplementary table 5). Patients who experienced SevEx only had better outcomes than those who experienced AWE only. When considered according to treatment group, the benefits of AQLQ and ACQ-6 were greatest among those who experienced SevEx only. However, a moderate benefit was observed for patients who experienced no CompEx events, AWE only or mixed events (figure 7).

## Discussion

In this *post hoc* analysis of CompEx events, patients who experienced AWE only or mixed events had greater deteriorations in mean morning and evening PEF, and increased symptom scores and reliever medication use pre- and post-event, compared with patients who experienced SevEx only; this was reflected by quality-of-life measures. Recovery was faster in patients experiencing SevEx as their first event (in those experiencing SevEx only) than among those experiencing AWE as their first event (in those experiencing AWE only) or those experiencing concomitant SevEx/AWE as their first event. Further research is required to gain a better understanding of the underlying biological factors contributing to delayed recovery in these participants, as well as to determine the most effective methods for providing support and managing their condition. The improvements seen in those with SevEx only probably relate to their treatment with SCS, with rapidity of improvement suggesting that patients may engage in anticipatory treatment-seeking based on their experience of prior events. This may also explain the relatively small deteriorations in PEF seen in our data. Patient behaviour and socioeconomic environments may be one of the reasons for this finding because of the use of systemic steroids as soon as an exacerbation is felt to be coming or the use of systemic steroids prophylactically owing to easy access to treatment. This can result in “smaller events” from which the patient recovers very quickly. The rate of anticipatory treatment-seeking behaviour may be difficult to reduce and could confound results from clinical trials of exacerbation-reducing treatments, due to the definition of the endpoint. Patients who experienced SevEx only could also have a better outcome because these events are not associated with objective measures of deterioration such as AWEs. They may also respond very well to systemic steroids.

Patients experiencing AWEs only may not have easy access to treatment or might be steroid averse, resulting in events that, although serious, are self-managed without steroids, leading to longer-term poorer health outcomes.

Nevertheless, these results demonstrate that AWEs are meaningful events that significantly contribute to poor lung function and patients who experience them have poorer outcomes over time (likely because they are treated differently than SevEx). Patients with a mixture of SevEx and AWE, including concomitant SevEx/AWE, had the poorest outcomes.

AWEs, which are not typically reported in clinical trials and are evaluated based on deteriorations in PEF accompanied by increased reliever medication use and/or worsening of symptoms, reflect multiple domains of the impact of asthma on patients. Furthermore, they may provide a more objective evaluation of asthma control than SevEx, defined based on SCS treatment, which depends on healthcare professionals’ perception of symptoms. By evaluating AWEs in addition to SevEx within a single composite endpoint, the different subtypes of CompEx events encompass all domains of disease activity.

When considered according to treatment group, compared with placebo, benralizumab was associated with improvements in FEV_1_ in patients with no CompEx events. When comparing the outcomes in the event groups with outcomes in the no-CompEx group, it is important to note that the event groups were weighted more towards placebo treatment than the no-CompEx group. Improvements in AQLQ and ACQ-6 trajectories, following benralizumab treatment, were seen in all CompEx subgroups, with the greatest benefits, compared with placebo, seen in patients experiencing SevEx only. Benralizumab did not reduce the risk of an event in the group that experienced AWEs only, potentially owing to different factors driving this type of event, which may not be due solely to eosinophilic inflammation. These events could, in part, be the result of events that would be SevEx but have had their severity decreased by treatment and are therefore eosinophilic in nature. However, it is unlikely that this is the cause of all AWEs as they are also observed in placebo-treated patients. These may therefore be non-eosinophilic (other dominant mechanisms of action) or may either be driven by patient behaviour or arise because the patient's personal threshold for healthcare-seeking behaviour is high and events that other patients consider severe are not recorded as such.

Limitations of this study relate to the *post hoc* nature of the analyses, including the non-randomised analysis and lack of prespecified subgroups. Also, the methodology for data collection was primarily reliant on the utilisation of patient electronic diary entries: although this was not an issue in the current study, it is important to mention that e-diary compliance needs to be ≥70% for results to be interpretable. A strength of this study was the use of a comprehensive dataset with a large number of patients from two trials with similar study designs and outcomes.

### Conclusion

These data suggest that AWEs are important contributors to poor asthma outcomes and should not be ignored when evaluating treatments. Therefore, the use of CompEx as an endpoint may support the evaluation of asthma therapies, as AWEs are not captured by SevEx criteria.

## Data Availability

Data underlying the findings described in this manuscript may be obtained in accordance with AstraZeneca's data-sharing policy described at https://astrazenecagrouptrials.pharmacm.com/ST/Submission/Disclosure.

## Supplementary material

**Please note:** supplementary material is not edited by the Editorial Office, and is uploaded as it has been supplied by the author. Supplementary material 00486-2025.SUPPLEMENT

## References

[1] Bourdin A, Bjermer L, Brightling C, et al. ERS/EAACI statement on severe exacerbations in asthma in adults: facts, priorities and key research questions. Eur Respir J 2019; 54: 1900900. doi:10.1183/13993003.00900-2019 31467120

[2] Larsson K, Kankaanranta H, Janson C, et al. Bringing asthma care into the twenty-first century. NPJ Prim Care Respir Med 2020; 30: 25. doi:10.1038/s41533-020-0182-2 32503985 PMC7275071

[3] Global Initiative for Asthma (GINA). Global Strategy for Asthma Management and Prevention. 2023. Date last accessed: 5 January 2024. https://ginasthma.org/2023-gina-main-report/

[4] National Heart, Lung, and Blood Institute (NHLBI). 2020 Focused Updates to the Asthma Management Guidelines. 2020. Date last accessed: 2 January 2024. www.nhlbi.nih.gov/health-topics/all-publications-and-resources/2020-focused-updates-asthma-management-guidelines

[5] Reddel HK, Taylor DR, Bateman ED, et al. American Thoracic Society/European Respiratory Society Task Force on Asthma Control and Exacerbations. An official American Thoracic Society/European Respiratory Society statement: asthma control and exacerbations: standardizing endpoints for clinical asthma trials and clinical practice. Am J Respir Crit Care Med 2009; 180: 59–99. doi:10.1164/rccm.200801-060ST 19535666

[6] Castro M, King TS, Kunselman SJ, et al. National Heart, Lung, and Blood Institute's AsthmaNet. Effect of vitamin D_3_ on asthma treatment failures in adults with symptomatic asthma and lower vitamin D levels: the VIDA randomized clinical trial. JAMA 2014; 311: 2083–2091. doi:10.1001/jama.2014.5052 24838406 PMC4217655

[7] Liu MC, Bel EH, Kornmann O, et al. Health outcomes after stopping long-term mepolizumab in severe eosinophilic asthma: COMET. ERJ Open Res 2022; 8: 00419-2021. doi:10.1183/23120541.00419-2021 35036420 PMC8752942

[8] FitzGerald JM, Hamelmann E, Kerstjens HAM, et al. Asthma exacerbations and worsenings in patients aged 1–75 years with add-on tiotropium treatment. NPJ Prim Care Respir Med 2020; 30: 38. doi:10.1038/s41533-020-00193-w 32868782 PMC7459309

[9] Sculpher MJ, Buxton MJ. The episode-free day as a composite measure of effectiveness: an illustrative economic evaluation of formoterol versus salbutamol in asthma therapy. Pharmacoeconomics 1993; 4: 345–352. doi:10.2165/00019053-199304050-00005 10146873

[10] Woodcock AA, Bagdonas A, Boonsawat W, et al. GOAL Steering Committee and Investigators. Improvement in asthma endpoints when aiming for total control: salmeterol/fluticasone propionate *versus* fluticasone propionate alone. Prim Care Respir J 2007; 16: 155–161. doi:10.3132/pcrj.2007.00043 17551661 PMC6634212

[11] Zhang J, Song C, Reiss TF. An endpoint for worsening asthma: development of a sensitive measure and its properties. Drug Inf J 2004; 38: 5–13. doi:10.1177/009286150403800103

[12] Fuhlbrigge AL, Bengtsson T, Peterson S, et al. A novel endpoint for exacerbations in asthma to accelerate clinical development: a *post hoc* analysis of randomised controlled trials. Lancet Respir Med 2017; 5: 577–590. doi:10.1016/S2213-2600(17)30218-7 28583396

[13] Jauhiainen A, Scheepers L, Fuhlbrigge AL, et al. Impact of season and geography on CompEx Asthma: a composite end-point for exacerbations. ERJ Open Res 2020; 6: 00246-2020. doi:10.1183/23120541.00246-2020 PMC7569167 33123561

[14] Bolton C, Harrison T, Lugogo N, et al. Use of CompEx in eosinophilic patients with severe, uncontrolled asthma on benralizumab. ERJ Open Res 2024; 10: 01025-2023. doi:10.1183/23120541.01025-2023 38500798 PMC10945385

[15] Bleecker ER, FitzGerald JM, Chanez P, et al. SIROCCO study investigators. Efficacy and safety of benralizumab for patients with severe asthma uncontrolled with high-dosage inhaled corticosteroids and long-acting beta(2)-agonists (SIROCCO): a randomised, multicentre, placebo-controlled phase 3 trial. Lancet 2016; 388: 2115–2127. doi:10.1016/S0140-6736(16)31324-1 27609408

[16] FitzGerald JM, Bleecker ER, Nair P, et al. CALIMA study investigators. Benralizumab, an anti-interleukin-5 receptor alpha monoclonal antibody, as add-on treatment for patients with severe, uncontrolled, eosinophilic asthma (CALIMA): a randomised, double-blind, placebo-controlled phase 3 trial. Lancet 2016; 388: 2128–2141. doi:10.1016/S0140-6736(16)31322-8 27609406

[17] AstraZeneca. Fasenra™ (Benralizumab). Prescribing information. Date last accessed: 11 July 2023. www.azpicentral.com/fasenra/fasenra_pi.pdf

