# Supplementary material for: Clinical relevance of CompEx Asthma and impact on disease trajectory: benralizumab effect
Source: ERJ Open Res. 2026 Feb 23;12(1):00486-2025. doi: 10.1183/23120541.00486-2025 (PMC12926827; doi:10.1183/23120541.00486-2025)
Supplement: Supplementary file 1 [file 00486-2025.SUPPLEMENT.pdf]

Supplementary material

Supplementary information

CompEx events were defined as occurrence of either [12]:

- A severe exacerbation defined as an asthma worsening leading to use of systemic corticosteroids, an emergency room visit and/or hospitalisation
- A diary event, defined as occurrence of at least two asthma worsenings:
  - 15% decline from baseline in morning or evening peak expiratory flow
  - 1.5x increase in nighttime or daytime reliever use
  - Increase in daytime or nighttime symptom score by 1 point from baseline or absolute maximum score
  - Deterioration over 5 days:
    - 3%/day decrease in peak expiratory flow
    - 0.3 dose/day increase in reliever use
    - 0.2 score/day increase in symptom score

Statistical methods

Region, previous exacerbations and systemic corticosteroid use were stratification variables and used in the randomization, and have been included in all statistical models. In the pooled analysis of the two studies, study has been added as an extra factor as is standard for meta analysis.

In the original manuscripts the assumption of proportional hazards was checked by testing the treatment-by-time interaction and graphically checking linearity. In these new analyses we maintained use of Cox models for consistency with previous results and proportionality was only checked visually using Kaplan–Meier plots.

Analyses of visit-based data (not in manuscript) were provided by two different models: an ANCOVA approach based on last value carried forward to handle missing data; and a mixed model repeated measures with no imputation. Graphical representations were based on imputed data using the last value carried forward approach and presented raw means.

This is a post hoc evaluation of data from two clinical studies and results should be seen as hypothesis generating rather than confirmatory. Thus, no adjustment for multiple tests were provided.

Sample size was originally provided for the original studies separately. In this post hoc evaluation we used the subset of patients with high eosinophils at baseline, either separately by study or pooled across the two studies. A post hoc calculation of power was not performed since the evaluation is hypothesis generating rather than confirmatory and looks at several aspects of the endpoints performance in CompEx subtypes. Thus a set of endpoints is used without identifying a primary endpoint to base a power calculation on.

Missing data were not imputed when calculating CompEx or AWEs. For other purposes, diary data were averaged by week, with a valid week including at least 4 days with data. Missing or not valid weekly averages were imputed using last observation carried forward as stated in the statistical section.

Supplementary figure 1. a) Morning (left) and evening (right) changes in PEF, day-time (left) and night-time (right) b) asthma symptoms and c) reliever medication use around the occurrence of a CompEx event in the combined CALIMA and SIROCCO trials according to treatment received. The three groups analysed were first SevEx in the group who experienced SevEx only, first AWE in the group who experienced AWEs only and concomitant SevEx/AWE when it was the first event experienced in the group reporting mixed events. The shaded region shows the 95% confidence intervals.

AWE: acute worsening event; CompEx: composite endpoint; PEF: peak expiratory flow; SevEx: severe exacerbation.

a)

Combined treatment arms

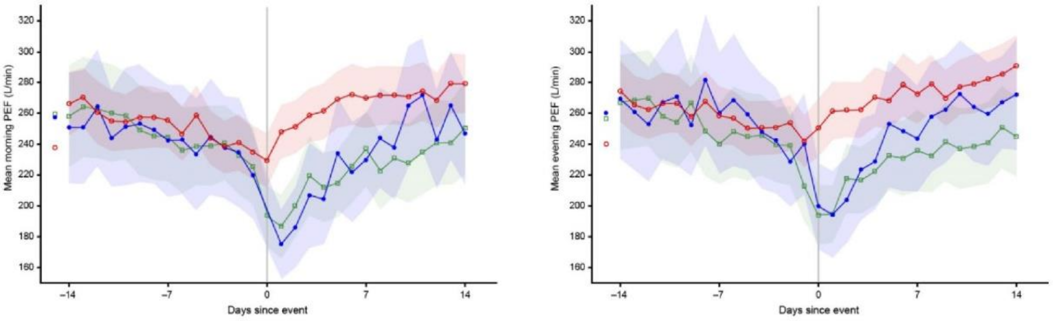

Separate treatment arms

Placebo

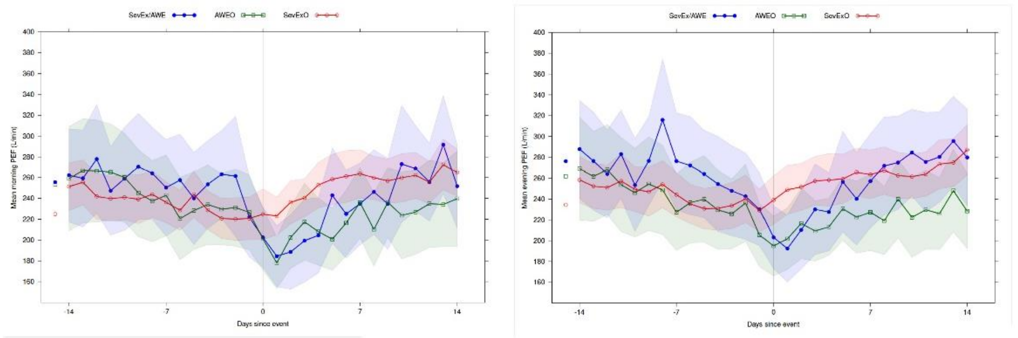

Benralizumab

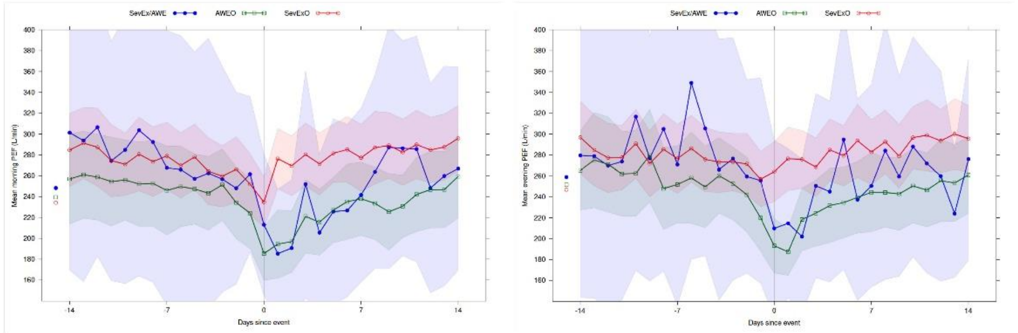

b)

Combined treatment arms

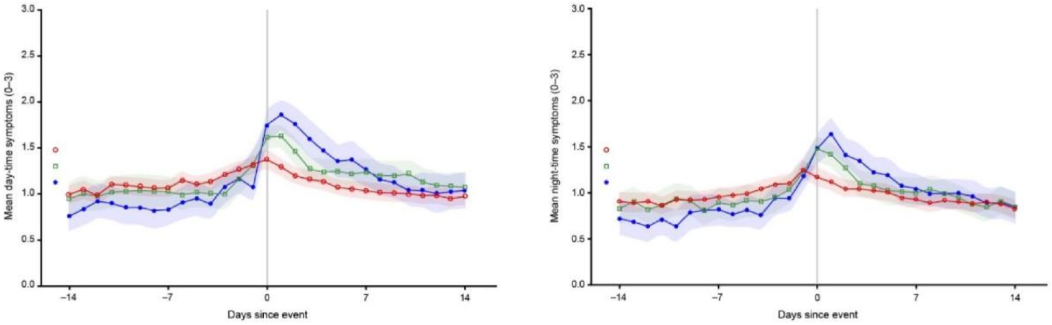

Separate treatment arms

Placebo

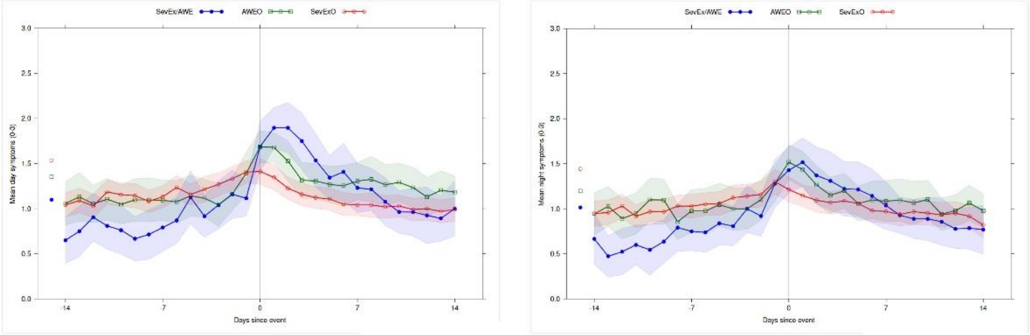

Benralizumab

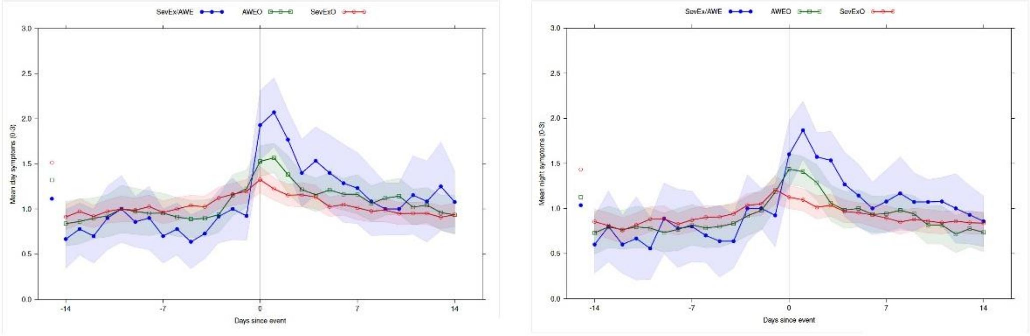

c)

Combined treatment arms

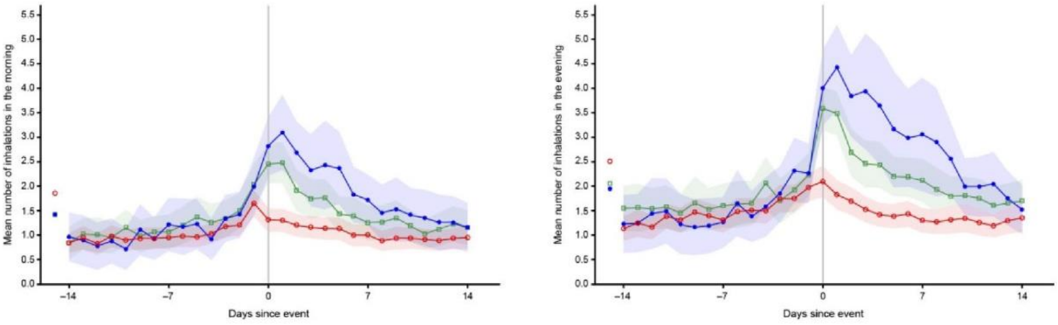

Separate treatment arms

Placebo

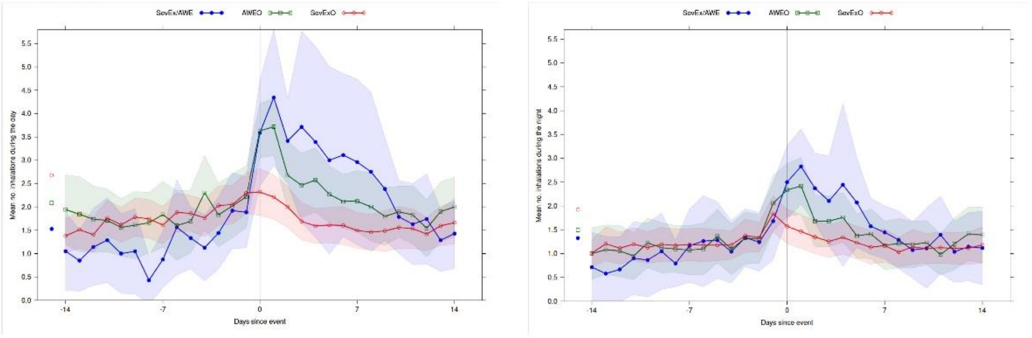

Benralizumab

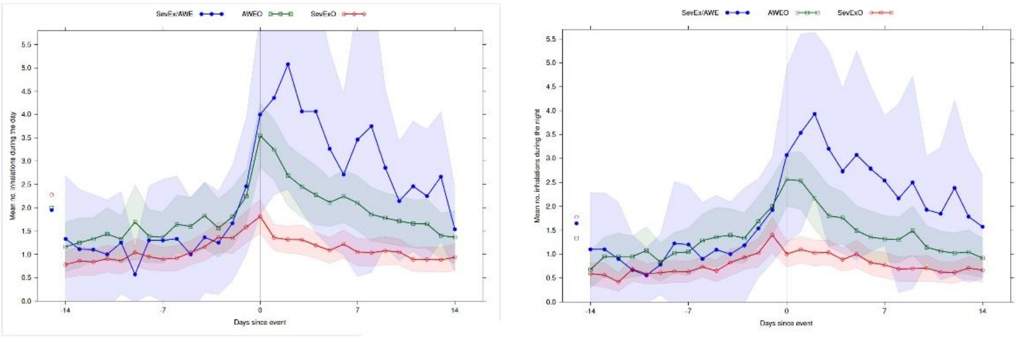

Supplementary figure 2. Recovery following a CompEx event to mean baseline values of a) morning (left) and evening (right) PEF, day-time (left) and night-time (right), b) asthma symptoms and c) reliever medication use in the combined CALIMA and SIROCCO trials according to treatment received. The three groups analysed were first SevEx in the group who experienced SevEx only, first AWE in the group who experienced AWEs only and concomitant SevEx/AWE when it was the first event experienced in the group reporting mixed events. Day 0 was the first day of the analysed event. AWE: acute worsening event; CompEx: composite endpoint; PEF: peak expiratory flow; SevEx: severe exacerbation.

a)

Combined treatment arms

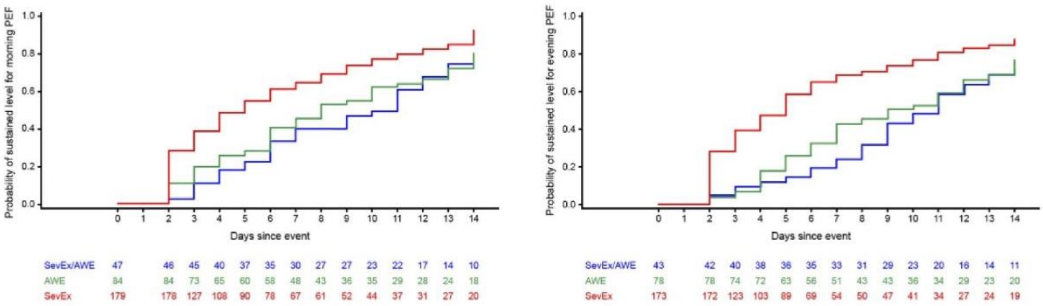

Separate treatment arms

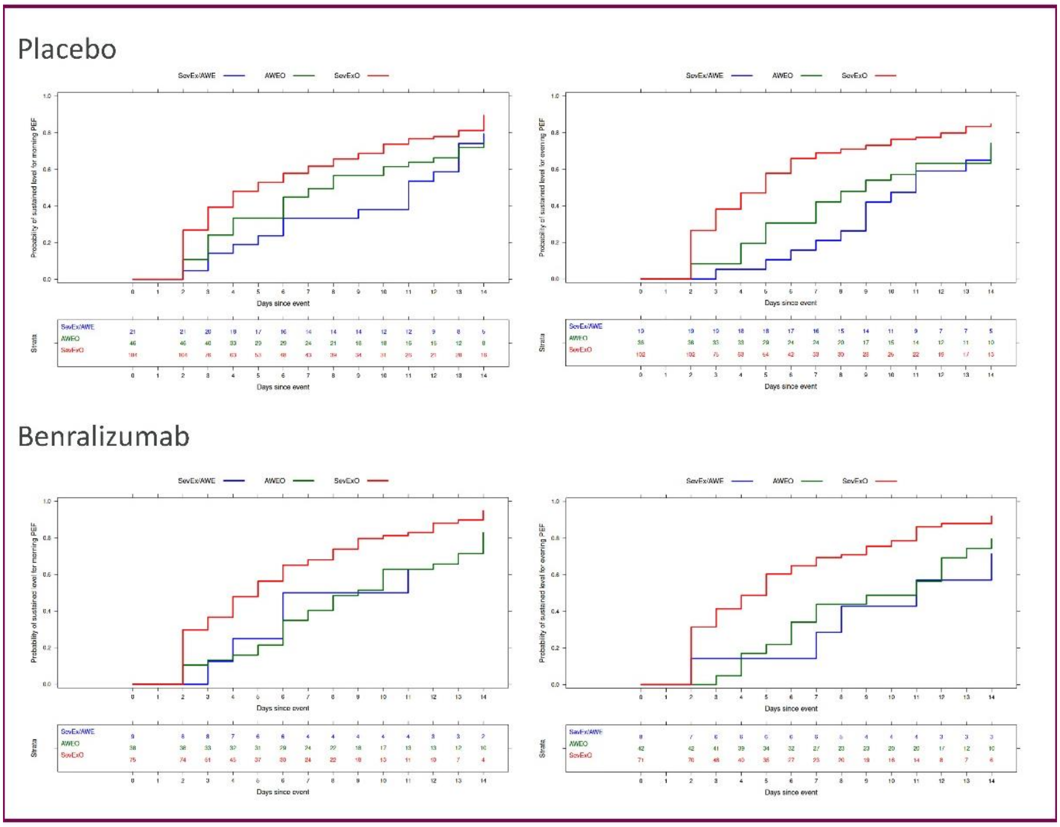

b)

Combined treatment arms

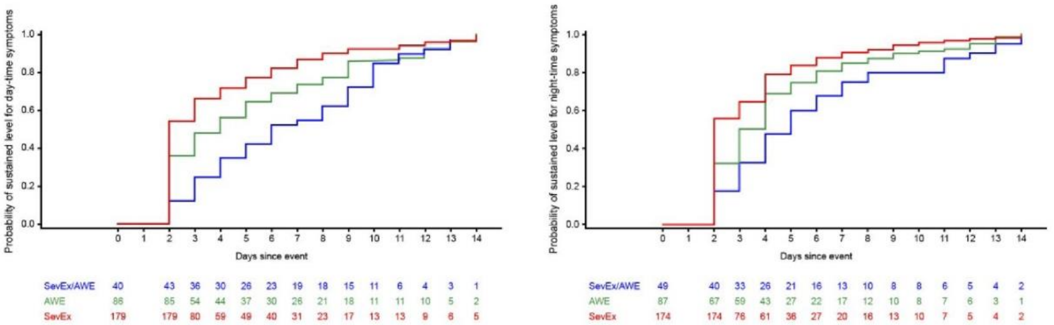

Separate treatment arms

Placebo

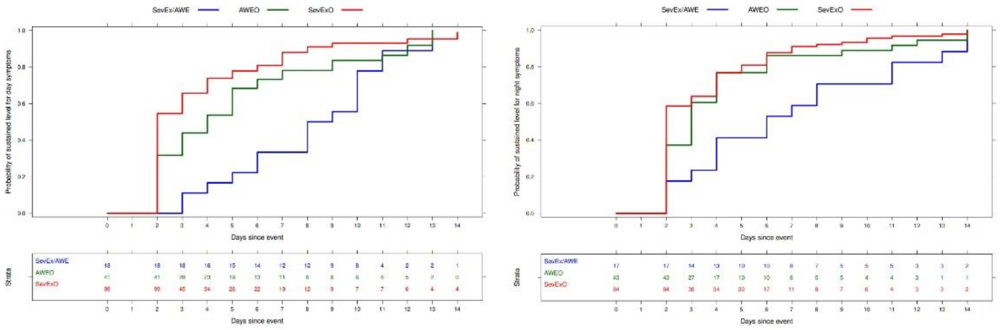

Benralizumab

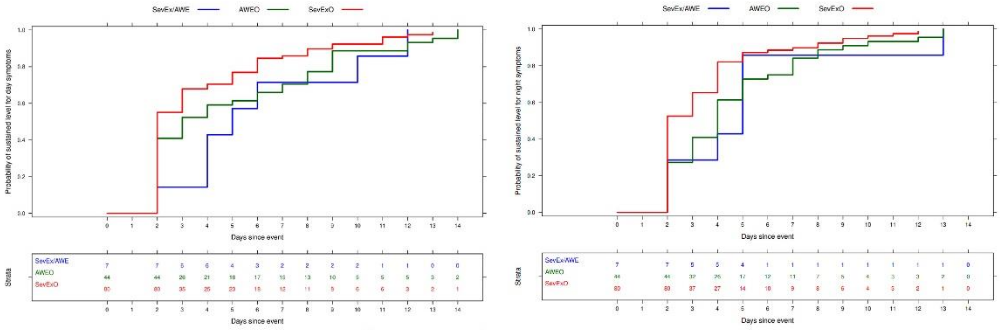

c)

Combined treatment arms

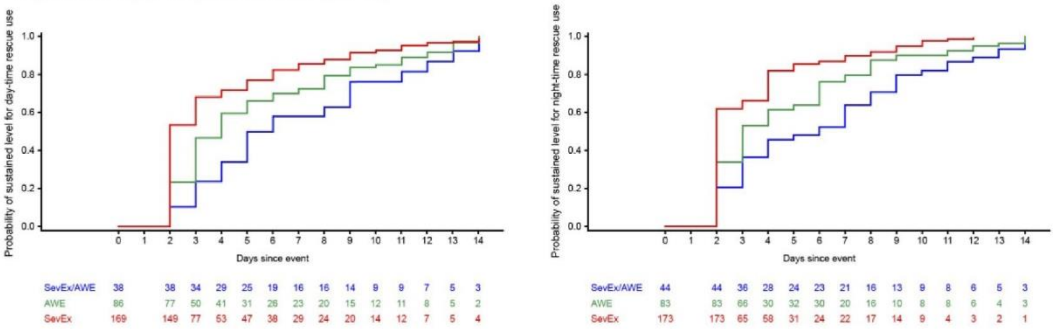

Separate treatment arms

Placebo

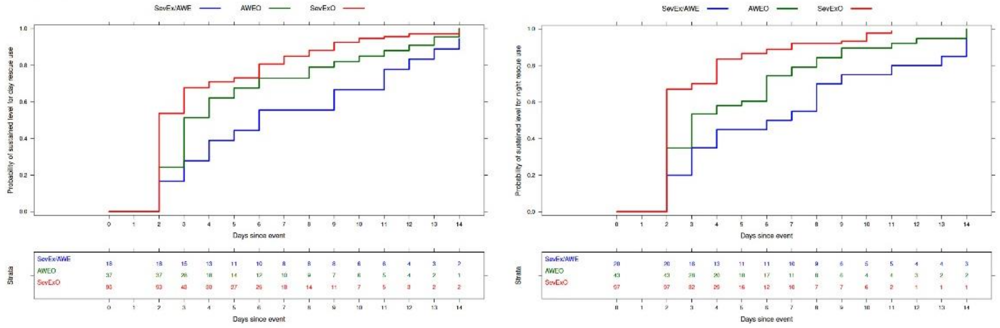

Benralizumab

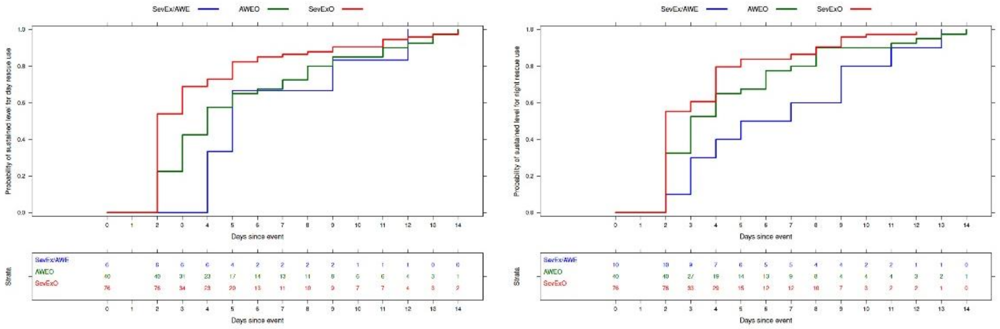

Supplementary table 1. Recovery following a CompEx event to the mean baseline values of morning and evening PEF, asthma symptoms and reliever medication use in the combined CALIMA and SIROCCO trials

Variable	Events	Hazard ratio	Lower CI	Upper CI	p-value	p-homogeneity
			**PEF**			
**Morning**	AWE only vs SevEx/AWE	1.11	0.72	1.73	0.633	<0.001
	SevEx only vs AWE only	1.82	1.34	2.48	<0.001	
	SevEx only vs SevEx/AWE	2.03	1.36	3.01	<0.001	
**Evening**	AWE only vs SevEx/AWE	1.08	0.71	1.64	0.715	<0.001
	SevEx only vs AWE only	1.62	1.21	2.18	0.001	
	SevEx only vs SevEx/AWE	1.75	1.21	2.53	0.003	
			**Asthma symptoms**			
**Night-time**	AWE only vs SevEx/AWE	1.35	0.93	1.98	0.118	0.007
	SevEx only vs AWE only	1.26	0.97	1.64	0.080	
	SevEx only vs SevEx/AWE	1.71	1.20	2.44	0.003	
**Day-time**	AWE only vs SevEx/AWE	1.30	0.89	1.90	0.182	0.005
	SevEx only vs AWE only	1.32	1.01	1.72	0.040	
	SevEx only vs SevEx/AWE	1.71	1.20	2.43	0.182	
			**Reliever medication use**			
**Night-time**	AWE only vs SevEx/AWE	1.38	0.95	1.99	0.092	<0.001
	SevEx only vs AWE only	1.38	1.06	1.80	0.018	
	SevEx only vs SevEx/AWE	1.90	1.35	2.68	<0.001	
**Day-time**	AWE only vs SevEx/AWE	1.34	0.90	1.99	0.144	0.002
	SevEx only vs AWE only	1.36	1.04	1.80	0.027	
	SevEx only vs SevEx/AWE	1.83	1.28	2.63	0.001	

Patients in the SevEx only group, experienced only SevEx throughout the trial. Patients in the AWE only group, experienced only AWE throughout the trial. Patients in the mixed events group experienced both AWE and SevEx during the trial, including concomitant SevEx/AWE. AWE, acute worsening event; CI, confidence interval; CompEx, composite endpoint; PEF, peak expiratory flow; SevEx, severe exacerbation.

Supplementary table 2. Benralizumab efficacy on the CompEx event types

Event	n	Hazard ratio	Lower CI	Upper CI	p-value
SevEx only	214	0.695	0.530	0.911	0.0084
AWE only	111	0.923	0.635	1.341	0.6741
Mixed events	204	0.635	0.466	0.866	0.0041

Patients in the SevEx only group, experienced only SevEx throughout the trial. Patients in the AWE only group, experienced only AWE throughout the trial. Patients in the mixed events group experienced both AWEs and SevEx during the trial, including concomitant SevEx/AWE. AWE, acute worsening event; CI, confidence interval; CompEx, composite endpoint; SevEx, severe exacerbation.

Supplementary table 3. Summary of tests of change from baseline in FEV_1 (L) compared with occurrence of CompEx events at weeks 24 and 48*

Week	Estimate	Lower CI	Upper CI	p-value
**SevEx only vs no CompEx**				
24	–0.1933	–0.2808	–0.1059	<0.0001
48	–0.1544	–0.2408	–0.06802	0.0005
**AWE only vs no CompEx**				
24	–0.1609	–0.2788	–0.04295	0.0076
48	–0.1190	–0.2366	–0.00148	0.0472
**Mixed events vs no CompEx**				
24	–0.2544	–0.3393	–0.1696	<0.0001
48	–0.1980	–0.2847	–0.1112	<0.0001

*ANCOVA analysis by patient subgroup and baseline. AWE, acute worsening event; CI, confidence interval; CompEx, composite endpoint; SevEx, severe exacerbation.

Supplementary table 4. Summary of tests of change from baseline in ACQ-6 compared with occurrence of CompEx events at weeks 24 and 48*

Week	Estimate	Lower CI	Upper CI	p-value
**SevEx only vs no CompEx**				
24	0.2212	0.04280	0.3996	0.0152
48	0.3002	0.1198	0.4807	0.0011
**AWE only vs no CompEx**				
24	0.4239	0.1975	0.6503	0.0003
48	0.6162	0.3831	0.8493	<.0001
**Mixed events vs no CompEx**				
24	0.6407	0.4623	0.8191	<.0001
48	0.8274	0.6460	1.0089	<.0001

*ANCOVA analysis by patient subgroup and baseline. ACQ-6, 6-item Asthma Control Questionnaire; AWE, acute worsening event; CI, confidence interval; CompEx, composite endpoint; SevEx, severe exacerbation.

Supplementary table 5. Summary of tests of change from baseline in AQLQ compared with occurrence of CompEx events at weeks 24 and 48*

Week	Estimate	Lower CI	Upper CI	p-value
**SevEx only vs no CompEx**				
24	–0.0638	–0.2580	0.1304	0.5191
48	–0.1591	–0.3584	0.0403	0.1176
**AWE only vs no CompEx**				
24	–0.2955	–0.5459	–0.0451	0.0208
48	–0.4187	–0.6782	–0.1592	0.0016
**Mixed events vs no CompEx**				
24	–0.5298	–0.7213	–0.3383	<0.0001
48	–0.6750	–0.8723	–0.4778	<0.0001

*ANCOVA analysis by patient subgroup and baseline. AQLQ, Asthma Quality of Life Questionnaire; AWE, acute worsening event; CI, confidence interval; CompEx, composite endpoint; SevEx, severe exacerbation.
